# Genome Mining Reveals High Biosynthetic Potential of Biocontrol Agent *Bacillus velezensis* B.BV10

**DOI:** 10.3390/genes13111984

**Published:** 2022-10-30

**Authors:** Rosiana Bertê, Gustavo Manoel Teixeira, João Paulo de Oliveira, Maria Luiza Abreu Nicoletto, Daniel Vieira da Silva, Guilherme Gonçalves de Godoy, Danilo Sipoli Sanches, Juliano Tadeu Vilela de Resende, Ulisses de Padua Pereira, Ulisses Nunes da Rocha, Admilton Gonçalves de Oliveira

**Affiliations:** 1 Department of Microbiology, Universidade Estadual de Londrina, Londrina 86057-970, PR, Brazil; 2 Computer Science Department, Universidade Tecnológica Federal do Paraná, Cornélio Procópio 86300000, PR, Brazil; 3 Department of Agronomy, Universidade Estadual de Londrina, Londrina 86057-970, PR, Brazil; 4 Department of Preventive Veterinary Medicine, Universidade Estadual de Londrina, Londrina 86057-970, PR, Brazil; 5 Department of Environmental Microbiology, Helmholtz Centre for Environmental Research–UFZ GmbH, 04318 Leipzig, Germany; 6 Laboratory of Electron Microscopy and Microanalysis, Universidade Estadual de Londrina, Londrina 86057-970, PR, Brazil

**Keywords:** plant growth-promoting bacteria, biocontrol, biosynthetic pathways, *B. velezensis*

## Abstract

The present study demonstrates the biocontrol potential of a plant growth-promoting bacterial strain using three different approaches: (i) an in vitro evaluation of antagonistic activity against important phytopathogenic fungi; (ii) an evaluation under greenhouse conditions with strawberry plants to assess the control of gray mold; and (iii) an in silico whole genome sequence mining to assign genetic features such as gene clusters or isolated genes to the strain activity. The in vitro assay showed that the B.BV10 strain presented antagonistic activity, inhibiting the mycelial growth in all the phytopathogenic fungi evaluated. The application of the *Bacillus velezensis* strain B.BV10 under greenhouse conditions reduced the presence of *Botrytis cinerea* and increased the mean fruit biomass. The genome of B.BV10 was estimated at 3,917,533 bp, with a GC content of 46.6% and 4088 coding DNA sequences, and was identified as *B. velezensis*. Biosynthetic gene clusters related to the synthesis of the molecules with antifungal activity were found in its genome. Genes related to the regulation/formation of biofilms, motility, and the important properties for the rhizospheric colonization were also found in the genome. The current study offers a comprehensive understanding of the genomic architecture and control activity of phytopathogenic fungi by the *B. velezensis* strain B.BV10 that may substantiate the industrialization of this strain in the future.

## 1. Introduction

Bacterial strains that efficiently colonize plant roots and promote plant growth by direct or indirect mechanisms are known as plant growth-promoting bacteria (PGPB) [1]. PGPB establish specific symbiotic interactions with plants and colonize the surfaces, the intercellular space, or the interior of cells without causing any damage. These bacterial strains could be used in biocontrol, bio-fertilization, and biostimulation to improve plant growth under harsh conditions [2]. *Bacillus* spp. are important rhizospheric bacteria that can facilitate plant growth and crop yields through various mechanisms, among which we highlight the root growth stimulation and biological control through the induction of a systemic resistance and/or the production of antimicrobial metabolites.

A better understanding of the genetic information within a bacterial strain is necessary for a precise species delimitation inside the *Bacillus subtilis* group and genome mining; consequently, whole genome sequencing is required for acquiring the genomic data on the potential biocontrol candidates [3]. In other studies, it has been reported that strains of the genus *Bacillus* can control gray mold [4,5,6]. In silico studies of strains with validated biological control capabilities are a great option to understand better the genetic machinery of the strain and its relationship with the biological control properties.

The antimicrobial metabolites produced by *Bacillus* spp. are highly diverse [7]. According to their biosynthetic pathways, these metabolites can be divided into three main classes: nonribosomal peptides (NRPs), polyketides (PKs), and ribosomally synthesized post-translationally modified peptides (RiPPs) [8]. Genomic-based tools for analyzing genes encoding antimicrobial secondary metabolites are broadly used for effective application studies [9,10] and, therefore, better support the development of new products based on biocontrol agents. Some researchers have reported the effect of *Bacillus* spp. in managing plant diseases and pests [11,12,13,14].

Finding the appropriate isolates of potential biocontrol agents is considerably complex. In addition to being effective, the biocontrol agent must also be convertible into efficient formulations that can be mass-produced and widely used [13]. In our study, the B.BV10 strain presented extremely important characteristics for industrialization, being a great differential, meaning it has an easy fermentation, a low need for formulation complexity, and the effective control of pathogens of agronomic importance. Therefore, the current study was designed to evaluate the potential of a plant growth-promoting bacterial strain using three different approaches: an in vitro evaluation of the antagonistic activity against important phytopathogenic fungi, an in vivo evaluation of the potential to control gray mold growth on strawberry plants under greenhouse conditions, and in silico whole genome sequence mining to assign genetic features such as gene clusters or isolated genes to the strain activity.

## 2. Materials and Methods

### 2.1. Plant-Pathogenic Fungi

Isolates of *Sclerotinia sclerotiorum*, *Macrophomina phaseolina*, *Fusarium oxysporum*, and *B. cinerea* were provided by Dra. Maria Isabel Balbi-Peña of the Plant Pathology Laboratory of the Department of Agronomy of the State University of Londrina (UEL). Dr. Artur Soares (Simbiose-Agro, Cruz Alta, RS, Brazil) provided an isolate of *Colletotrichum truncatum.* The isolates were grown on potato dextrose agar (PDA) (Neogen Corporation, Lansing, MI, USA) in 90 mm × 10 mm polystyrene plates and incubated for 7 days at 25 ± 1 °C, with 12 h of fluorescent light and 12 h of darkness. All isolates were deposited in the microbial culture collection of the Laboratory of Microbial Biotechnology—LABIM, UEL, Londrina, Brazil.

### 2.2. Origin of Isolate B.BV10, Phenotypic Characterization, and Colony Architecture

The isolate B.BV10 was sent to LABIM by Dr. Artur Soares from the company Simbiose-Agro, Santa Helena, Brazil, for genomic studies through a research project (Project No. 433-FAUEL/UEL, Londrina, Brazil). Gram staining was conducted using a Gram-stain kit for morphological visualization and cell wall definition. The endospore formation was observed using the Wirtz–Conklin method. Scanning electron microscopy (SEM) was carried out to visualize the colony morphology. In this step, a colony grown on agar for 48 h was removed and fixed in a solution containing 2.5% glutaraldehyde and 2% paraformaldehyde in 0.1 M of sodium cacodylate buffer (pH 7.2) at 4 °C. The samples were maintained in this solution overnight for fixation, after which they were washed three times with 0.1 M of sodium cacodylate buffer (pH 7.2) for 10 min, followed by dehydration three times for 10 min in an ethanol series (30, 50, 70, 90, and 100%). The samples were then submitted to critical point drying with CO_2_ (BALTEC CPD 030 Critical Point Drier), coated with gold (BALTEC SDC 050 Sputter Coater), and observed with an FEI Quanta 200 scanning electron microscope operating at 25.0 kV.

### 2.3. Dual Culture Assay

For the antifungal activity analysis, dual culture assays were carried out. For the strain activation, the isolate B.BV10 was cultured in LBA (Luria Bertani Agar, Neogen Corporation, Lansing, MI, USA) at 28 °C for 24 h. Next, B.BV10 was inoculated by continuous streaking 1 cm from the edge of the plates containing the PDA medium. A 6 mm mycelial plug taken from the edge of actively growing colonies of five phytopathogenic fungi (*S. sclerotiorum, M. phaseolina, F. oxysporum, B. cinerea*, and *C. truncatum*) was placed 1 cm from the edge on the opposite side. The experiment was incubated at 25 °C with a 12 h/12 h photoperiod for 7 days. For comparison purposes, a positive control was used containing the mycelial disc alone at 1 cm from the edge of the Petri dish, which was incubated under the same conditions. The growth was calculated, and the growth inhibition was determined using the following formula:

$$MGI\;\left(\%\right)=\left[\frac{\left(C-T\right)}{C}\right]\times 100$$

where *MGI* (%) is the percentage of the mycelial growth inhibition; *C* represents the colony radius of the fungal control plates; and *T* is the radius of the fungal colony in the treatment plates [14]. The experiment was repeated twice with 4 replicates, and the results were submitted to a one-way analysis of variance (ANOVA), and the means were compared using the Tukey test (*p* < 0.05).

### 2.4. In Vivo Biocontrol

In order to prepare the treatments with the B.BV10 isolate, the strain was activated on Luria-Bertani agar (LBA) (Neogen Corporation, Lansing, MI, USA) and incubated at 28 °C for 24 h. For the preparation of the pre-inoculum, the colonies were suspended in saline solution (0.85% sodium chloride, *w*/*v*), and the concentration was adjusted according to the 0.5 McFarland scale until reaching approximately 1.5 × 10^8^ CFU/mL. For the preparation of the inoculum, 30 µL of pre-inoculum were inoculated separately in 125 mL Erlenmeyer flasks containing 30 mL of culture medium containing (per Liter) 20.0 g of glucose; 12.4 g of tryptone; 5.0 g of NaCl; 1.5 g of K_2_HPO_4_ · 3H_2_O; 0.04 g of MnSO_4_ · H_2_O; 1.67 g of FeSO_4_ · 7H_2_O; and 1.22 g of MgCl_2_ · 6H_2_O with a pH 7.1, and incubated at 28 °C for 24 h at 125 RPM (Orbital shaker—Thoth 6430B, Piracicaba, SP, Brazil). For the fermentation, 4 mL of the inoculum were added to 400 mL of the same medium in 1000 mL Erlenmeyer flasks and incubated for 72 h at 28 °C and 200 RPM. The content of the flasks was frozen at −80 °C and lyophilized at −60 °C to obtain a powder containing B.BV10 metabolites and spores at 1 × 10^10^ CFU/g. The obtained lyophilic was used as an active ingredient for preparing a prototype product in the wettable powder presentation. For the formulation, the proportions of 25% of the lyophilic from the fermentation of B.BV10 and 75% of other inert ingredients were used. This prototype was used to prepare the different treatments, which varied in concentration: 1 g/L, 2 g/L, and 4 g/L in water.

The in vivo biocontrol experiment was carried out at the Fazenda Escola, UEL. The area is located at coordinates 23° 17′ S and 51° 10′ W and at an altitude of 570 m. The local climate is classified as mesothermal humid subtropical (Cfa) by the Köppen classification, with hot summers and moderate winters [15]. The local soil is classified as Eutroferric Red Latosol and has a clayey texture [16]. Seedlings of the San Andreas cultivar were acquired from importing companies in Chile. The bare root seedlings were transplanted into 64-cell polypropylene trays and filled with a commercial substrate based on biostabilized pine bark. The trays were kept in a greenhouse at a temperature of 27 ± 5 °C and a relative humidity of 80 ± 5%. For setting and rooting, foliar fertilizers were applied every 10 days. Pest and disease control was carried out preventively. The seedlings were kept for 30 days until formation and transplanted to the experimental field in July 2020. The test was implemented in a greenhouse in beds measuring 1.40 m in width, 49.6 m in length, and 15 cm in height, and coated with plastic film (double-sided mulching) measuring 25 microns thick. The soil was turned using a soil tiller and, based on soil chemical analysis, was fertilized with 130.3 kg/ha of dolomitic limestone to promote a slight increase in the pH value; the base fertilization consisted of 857.14 kg/ha of magnesium thermophosphate. In each bed, four rows of cultivation were implanted, with the holes alternating between one row and another, making a triangular shape. The seedlings were planted with a 30 cm spacing. The irrigation system adopted was a drip irrigation, with hoses provided with drippers spaced 15 cm apart (four lines of drip tapes per bed, each corresponding to a planting line). Topdressing fertilizations were carried out using fertigation. Every other week, 70 g per 1000 plants of a formulated fertilizer containing 6% N, 12% P_2_O_5_, 36% K_2_O, 1.8% Mg, and 8% S (Kristalon^®^) were used. In the intervention weeks, 70 g of calcium nitrate (Calcinit^®^) were used per 1000 plants, composed of 15.5% N and 19% Ca.

The experiment was implemented in randomized blocks, with six treatments and three replications, totaling eighteen plots. Each plot was 90 cm long and 80 cm wide, containing 12 plants. The treatments were as follows: T1—water; T2—fluazinam (Frowncide^®^ 500—Commercial synthetic chemical control); T3—*Bacillus amyloliquefaciens* D-747 (Eco shot^®^—commercial biological control); T4—B.BV10 1 g/L; T5—B.BV10 2 g/L; and T6—B.BV10 4 g/L. The commercial synthetic chemical and biological positive controls were prepared as recommended on the label. The treatments were started with the plants in the fruiting stage, with the natural presence of *B. cinerea* (gray mold). The treatments were applied every 7 days for 70 days, totaling 6 applications, between 8 and 10 am. The biological agents and commercial products were solubilized in 1 L of water and applied using a hand sprayer. A volume of 0.2 mL of BAIC^®^ active fixer was added per liter to improve the product’s effectiveness by enhancing the adhesion and breaking the surface tension of the water. The plants were sprayed until a surface runoff was achieved. The treatments were applied weekly for 70 days, with evaluations every 14 days, including the day of the beginning of the experiment, totaling 6 evaluations (0, 14, 28, 42, 56, and 70 days). In the analyses, the fruits that presented at least a 75% red epidermis were harvested and separated into healthy fruits and infected fruits.

After harvesting, the fruits were counted and weighed using a precision scale to the third decimal place. At the end of the productive period, the following variables were estimated: (a) the TFM: total fruit mass; (b) IFM: infected fruit mass; and (c) HFM: healthy fruit mass. Based on the IFM values, the AUDPC (area under the disease progress curve) was obtained. Data were submitted to the Bartlett, Durbin–Watson, and Shapiro–Wilk tests to verify if the assumptions of homogeneity, independence, and the normal distribution of the residuals were satisfied, respectively. In order to calculate the AUDPC, the EPIFITTER package was used [17]. All the data were submitted to a one-way ANOVA and the Scott–Knott test (*p* < 0.05).

### 2.5. Complete Genome Sequencing and Assembly

Aiming at a better understanding of the genetic information contained within the B.BV10 genome, its whole genome was sequenced. Starting from the stock tube, B.BV10 was activated in a nutrient agar plate, incubated at 28 °C for 48 h, and a single colony was selected for the DNA isolation. The Quick-DNA Miniprep Kit (Zymo Research, Irvine, CA, USA) was used for a genomic DNA extraction, and the library was assembled using the Nextera XT DNA library preparation kit. Isolate B.BV10 was sequenced using the MiSeq platform (BPI-Biotechnology Research and Innovation, Brazil) with the MiSeq Reagent V2 Micro (300 cycles, Illumina, San Diego, CA, USA). The quality of the reads was observed using FastQC, and the trimming parameters were applied using Trimmomatic [18], setting a threshold Phred score of 30, with several trimming parameters to obtain the best data possible for the assembly. The genome assemblies were performed using the IDBA-hybrid software [19], using the *B. velezensis* FZB42 genome as a reference for the read alignment. The generated contigs were aligned with a reference genome using the CONTIGuator software [20]; the raw reads from the sequencing were mapped against the generated scaffolds [21]; and the ones with low read counts to support the sequence were discarded. The gaps within the scaffold were first treated with the GapCloser tool [22], followed by a manual curation with reading mapping using a Bowtie2 and gap-filling using CLC Genomics Workbench 11 GUI (Qiagen, Germantown, MD, USA). The genome start was determined by a comparison with the reference strain *B. velezensis* FZB42, considering the dnaA gene as the first gene. The genome annotation was carried out using the RAST platform [23], where the CDSs were predicted and classified into subsystems. We used the PGAP pipeline for the GenBank deposit.

### 2.6. Phylogenomic Comparison and Tree

In order to precisely determine the isolate species using the isolate’s whole genome, OrthoANI (Orthologous Average nucleotide Identity) and dDDH (digital DNA-DNA hybridization) among all strains used as the reference sequences were determined using the OAT software [24] and the Genome-to-Genome Distance Calculator (GGDC) [25], respectively. We used the Gegenees software to make whole-genome comparisons between B.BV10 and the reference sequence strains [26]. Data from Gegenees were exported to SplitsTree for tree confection using the UPGMA method [27].

### 2.7. Representation of the Circularized Genome, Secondary Metabolite Cluster, and Colonization-Related Genes

The antiSMASH webserver combines different databases of genetic data, antimicrobial molecules, and biosynthesis gene clusters (BGCs) to predict the clusters’ position and possible function [28]. The analysis was carried out using the final FASTA file of B.BV10. The genome of strain B.BV10 and the BGCs were represented circularly and compared with other reference genomes (Table 1) using the BRIG (BLAST Ring Image Generator) software [29]. Data on the colonization-related sequences were obtained from the SubtiWiki repository [30], and the information was compiled into a multiFASTA file and compared with the complete genome of B.BV10.

### 2.8. Data Availability

The genome analyzed during the current study is available in DDBJ/EMBL/GenBank under the accession number CP059318.1 (https://www.ncbi.nlm.nih.gov/nuccore/NZ_CP059318.1, accessed on 1 August 2022) (BioProject PRJNA224116, (https://www.ncbi.nlm.nih.gov/bioproject/PRJNA224116, accessed on 1 August 2022) BioSample SAMN15484393 (https://www.ncbi.nlm.nih.gov/biosample/SAMN15484393, accessed on 1 August 2022)).

## 3. Results

### 3.1. Strain Characterization

**Figure 1:**
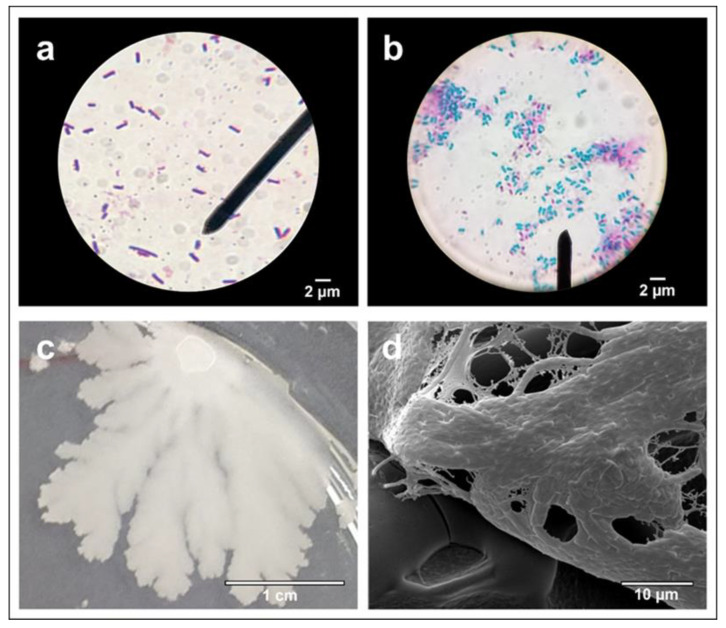
Micrograph of B.BV10 (100× magnification) under an optical microscope after Gram (**a**) and Wirtz–Conklin (**b**) staining; B.BV10 in LBA after 16 h of incubation (**c**); micrograph of B.BV10 (1000× magnification) in SEM (**d**).

The staining assays revealed that B.BV10 is a Gram-positive, rod-shaped bacterium (Figure 1a) capable of forming endospores (Figure 1b) [31]. The colony of B.BV10 has a cream color, is flattened, and has a smooth surface and irregular edges (Figure 1c). An observation of the colony by SEM showed that the cells of this strain aggregate with each other through the secretion of an extracellular matrix (Figure 1d) [32,33].

### 3.2. Dual Culture Assay

**Figure 2:**
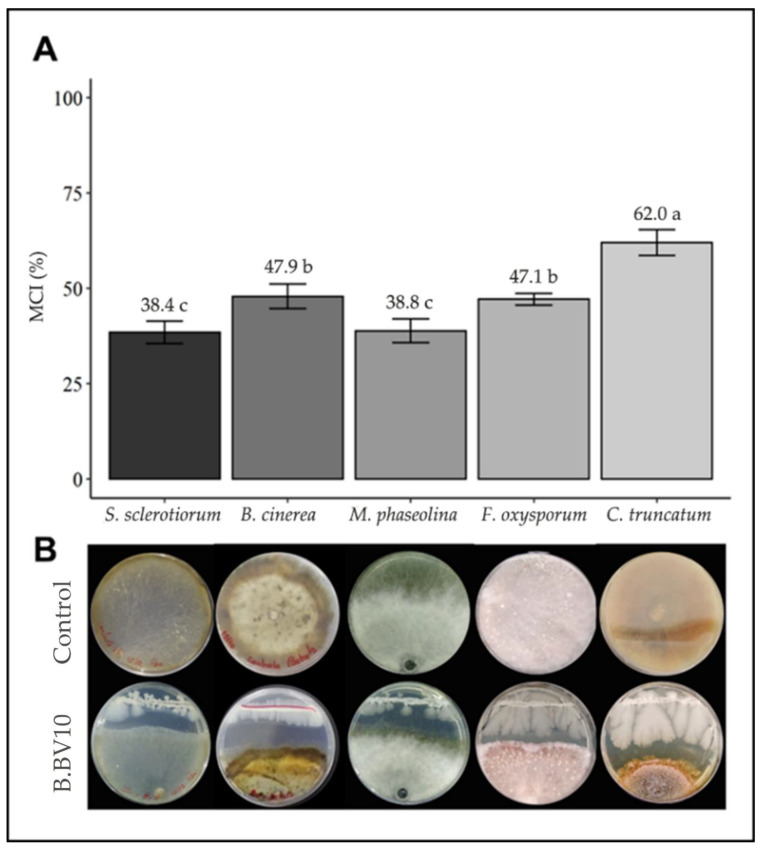
Antagonistic activity of B.BV10 against phytopathogenic fungi. MGI averages (%) were plotted into column charts (**A**), and below are the respective images of the in vitro assays (**B**). Means in each bar followed by the same letter did not differ significantly from each other by the Tukey test (*p* < 0.05).

The *B. velezensis* B.BV10 strain showed an antagonistic capacity in the dual culture assay, reducing the mycelial growth of all tested fungi (Figure 2A). It was possible to see that there was no direct contact between the two microorganisms (Figure 2B), which is a mechanism of antagonism related to the production of metabolites with antimicrobial activity [34]. The obtained MGI results were approximately 38% for *M. phaseolina* and *S. sclerotiorum*, 47% for *B. cinerea* and *F. oxysporum*, and 62% for *C. truncatum*, with statistically significant differences between each percentage.

### 3.3. In Vivo Biocontrol

**Figure 3:**
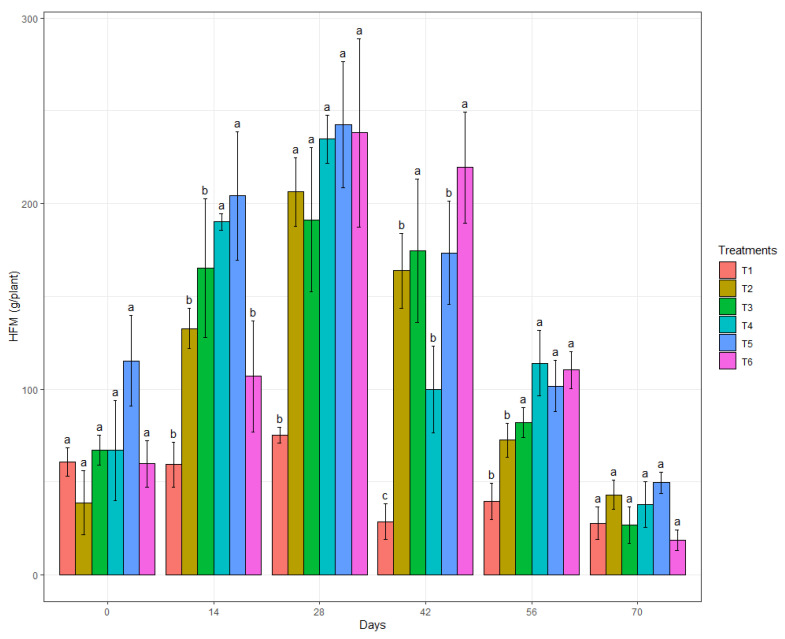
Mean HFM of the treatments (T1: water; T2: chemical control; T3: biological control; T4: B.BV10 1 g/L; T5: B.BV10 2 g/L; and T6: B.BV10 4 g/L) at each time of evaluation. Means in each day followed by the same letter did not differ significantly from each other by the Scott–Knott test (*p* < 0.05).

The HFM showed no differences among the treatments applied in the first and last evaluations (Figure 3). At 14 days after the first evaluation (DAFE), the highest HFM values were obtained in the treatments with B.BV10 at 1 g/L and 2 g/L. At 28 DAFE, there was an increase in the HFM in all treatments, which were significantly different from the water treatment. After 42 DAFE, the highest HFM was found in the biological control and B.BV10 treatments at a dose of 4 g/L. In the evaluation at 56 DAFE, a greater HFM was observed in all the biological treatments, while the smallest values were recorded in the treatments with water and chemical fungicide.

**Figure 4:**
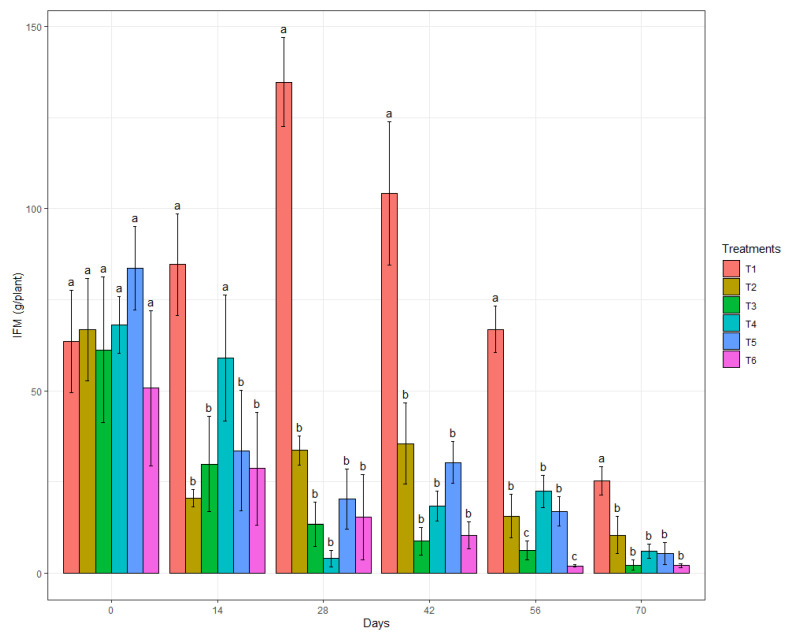
Mean IFM of the treatments (T1: water; T2: chemical control; T3: biological control; T4: B.BV10 1 g/L; T5: B.BV10 2 g/L; and T6: B.BV10 4 g/L) at each time of evaluation. Means in each day followed by the same letter did not differ significantly from each other by the Scott–Knott test (*p* < 0.05).

The presence of *B. cinerea* in the treatments did not differ significantly on the first day of the evaluation (Figure 4), which was expected due to the lack of a previous treatment of the strawberry plants. However, in the subsequent analyses, detecting a difference in the infected fruits among the treatments was possible. At 14 DAFE, only the treatment with B.BV10 at a dose of 1 g/L showed no difference in the IFM with the water treatment. At 28 DAFE, all treatments had significantly different IFM values compared to the water treatment, a trend that was maintained until the end of the experiment, except for the evaluation at 56 DAFE, in which the biological treatments with EcoShot^®^ and B.BV10 at a dose of 4 g/L were even more effective in reducing the IFM compared to the other treatments.

**Figure 5:**
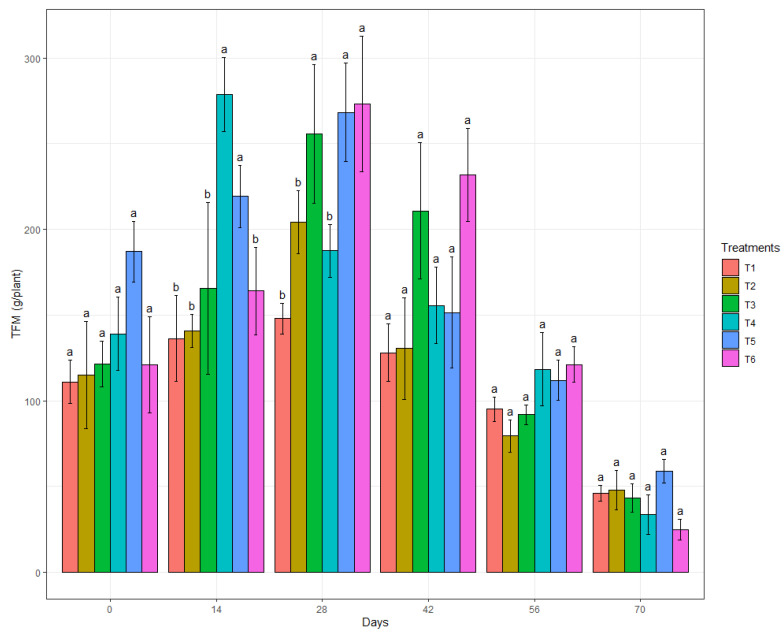
Mean TFM of the treatments (T1: water; T2: chemical control; T3: biological control; T4: B.BV10 1 g/L; T5: B.BV10 2 g/L; and T6: B.BV10 4 g/L) at each time of evaluation. Means in each day followed by the same letter did not differ significantly from each other by the Scott–Knott test (*p* < 0.05).

Overall, the TFM did not differ significantly among the treatments at most assessment times (Figure 5), except for the analyses at 14 and 28 DAFE, in which the biological treatments with B.BV10 at doses of 1 g/L and 2 g/L and EcoShot^®^ (only in 28 DAFE) showed higher TFM values. However, in the subsequent evaluations, no difference in the TFM was detected among the treatments. By also encompassing the IFM, the TFM data are not ideal for evaluating the productivity of strawberry plants. Nevertheless, throughout the experiment, there was a tendency for the TFM to be similar among the treatments, with significant differences in the IFM and HFM analyses.

**Figure 6:**
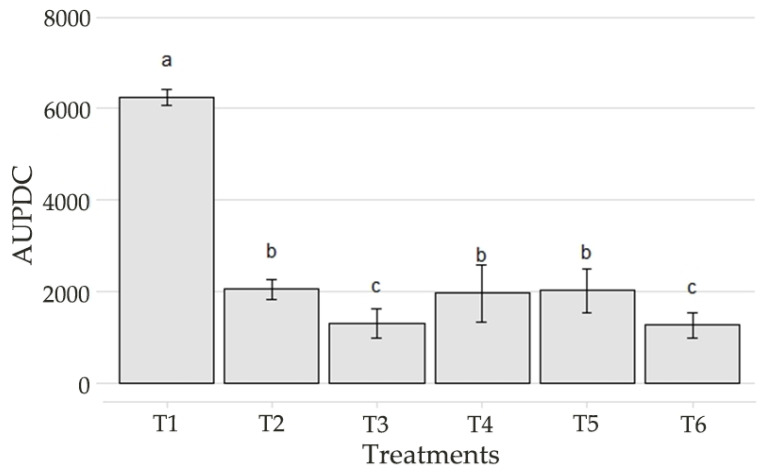
AUPDC of IFM data for each treatment (T1: water; T2: chemical control; T3: biological control; T4: B.BV10 1 g/L; T5: B.BV10 2 g/L; and T6: B.BV10 4 g/L). Means ± SE followed by the same letter did not differ significantly from each other by the Scott–Knott test (*p* < 0.05).

Based on the AUDPC data (Figure 6), it was possible to observe three situations: in the strawberry plants treated only with water, there was a higher incidence of *B. cinerea*, which led to a very high AUDPC and significant differences from the other treatments, indicating that treatments T2 to T6 could control the disease; the fungicides used as the controls showed different performances, with the biological fungicide exhibiting the best control effect; the treatment with B.BV10 at a dose of 4 g/L performed better than the treatments containing the strain at lower doses, indicating a better control effect when increasing the dose of the product, being superior to the chemical fungicide and similar to the biological fungicide.

### 3.4. Complete Genome Sequencing and Assembly

The sequencing and assembly of B.BV10 resulted in a genome with 3,917,533 bp, with a total alignment rate of 98.27% and a GC content of 46.6%. The genome annotation was performed using the PGAP pipeline, which generated data on the genome characteristics (Table 2).

### 3.5. Phylogenomic Comparison and Tree

**Figure 7:**
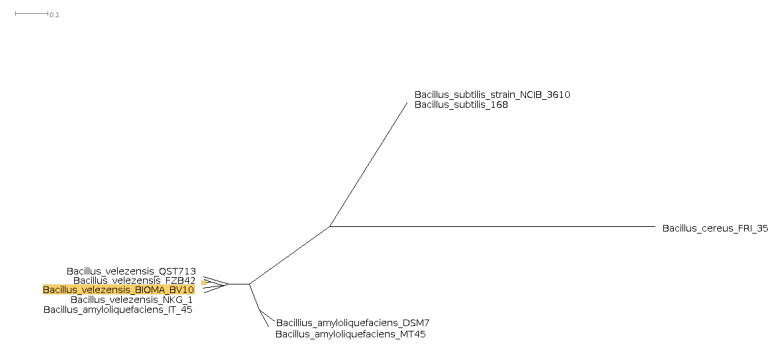
Phylogenomic tree representing the similarity between *B. velezensis* B.BV10 and strains from the *B. subtilis* group. The matrix was generated by Gegenees and exported to SplitsTree4 for making the tree using the UPGMA method.

Compared with the reference sequences of the genus *Bacillus*, using the OrthoANI, dDDH, and GGDC methods (Table 3), the strain B.BV10 showed a greater similarity with *B. velezensis* FZB42, with values of 99.26%, 94.30%, and 0.03, respectively. Based on the results and the tree organization (Figure 7), the B.BV10 strain was classified as *B. velezensis*.

### 3.6. Representation of the Circularized Genome, Secondary Metabolite Cluster, and Colonization-Related Genes

**Figure 8:**
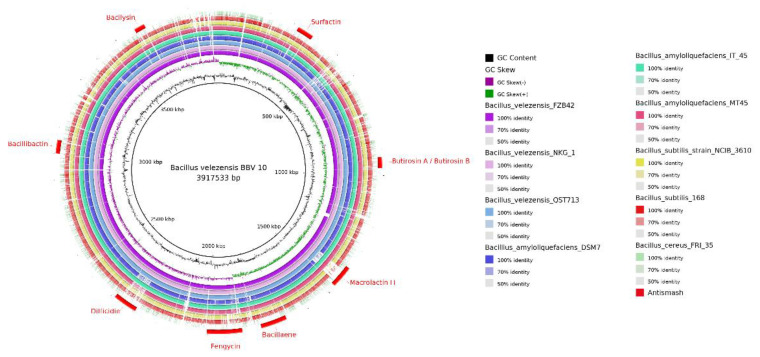
Circular representation of the genome of *B. velezensis* B.BV10 generated in the BRIG program. Inside-out matches, GC content, GC skew, FZB42, NKG1, QST713, DSM7, IT45, MT45, NCIB 3610, 168, FRI35, and position of BGCs in the genome indicated by antiSMASH.

AntiSMASH analysis found 12 BGCs of secondary metabolites in the *B. velezensis* B.BV10 genome (Table 4). The BGCs found were also represented in a circular genome (Figure 8), comparing the biosynthesis regions with the organisms selected for comparative analyses. Of the 12 clusters found, 8 showed a similarity with the BGCs deposited and described in the MiBiG repository [35], which are associated with the synthesis of surfactins (91% and similarity with the clusters known by antiSMASH), butyrosine A/B (7%), macrolactin H (100%), bacillaene (100%), fengycin (100%), difficidin (100%), and bacillibactin (100%); the other clusters showed no significant similarity with the clusters deposited in the repository.

Genes related to the colonization capacity were found in the *B. velezensis* B.BV10 genome (Table 5). Among the genes found are the surfactin operon sfrAA-AC, related to the regulation and production of surfactins, the epsA-O operon, related to exopolysaccharide expression, and the yqxM-sipW-tasA operon. This last operon is associated with the production of amyloid fibers, one of the components responsible for the union and cellular organization of the organisms in biofilms, and the swrA-C operon. The swrA-C operon is responsible for the expression of flagella and the swarming capacity of the strain. The genetic machinery and growth patterns of B.BV10 substantiate its strong colonizing ability. In this context, the sum of the in silico observations, together with the antagonistic activity data, allows us to infer that B.BV10 can protect crops against important phytopathogenic soil fungi and can be used as an active ingredient for the production of commercial biofungicides.

## 4. Discussion

The B.BV10 strain has essential properties as a biological control agent. Endospore formation is a characteristic of great interest to the industry since endospores increase the stability of the formulated biological products [31]. Furthermore, the secretion of the extracellular matrix observed in the SEM image of the colony has already been associated with biofilm production, an important characteristic related to plant colonization [14,32,33]. However, the main feature of the B.BV10 strain observed in this study was its biocontrol ability against phytopathogenic fungi of an agricultural importance, which had their growth inhibited in the in vitro experiments. In addition, under greenhouse conditions, the B.BV10 strain was able to control the growth of *B. cinerea*. These observations suggest the production of fungicidal biomolecules during the submerged liquid fermentation state and in the natural environment [14,34].

*B. cinerea* is responsible for causing gray mold in several crops, including strawberries. In strawberries, this fungus is particularly a problem during cultivation and also post-harvest [36]. The BBV10 strain showed biocontrol properties against *B. cinerea*, with a significant difference compared to the controls, demonstrating the great potential for elaborating a fungicidal product for use in strawberries, as well as in other cultures. Despite the excellent control effect on *B. cinerea* observed under greenhouse conditions, it is important to point out that no previous studies were carried out on the development of the fermentation processes and prototyping for the B.BV10 strain. Even so, the results show that the strain is a promising biocontrol agent for gray mold, with a similar efficacy to the biofungicide EcoShot^®^ and superior to the chemical-synthetic fungicide Frowncide^®^, two products widely used in Brazil. In this sense, new studies aimed at developing bioprocesses for B.BV10 could further increase its performance as a biofungicide for gray mold control. Allied with the fact that the production of strawberries has grown over the years all over the world, increasing in economic value, a product that controls a disease of considerable impact for this culture and also enables a greater productivity of healthy fruits for later marketing is of substantial interest to producers [37].

In this context, the B.BV10 strain proved efficient in both parameters, as it adequately controlled the incidence of gray mold while increasing the productivity of healthy fruits in the strawberries treated with the strain. In another study, using *B. velezensis* strain 83, the authors observed an increase in tomato production after treatment with the strain [38]. Meanwhile, in other studies with *Bacillus* ssp. strains, the ability to control gray mold was detected in grape, tomato, and pepper production [39,40,41]. In the present study, both traits were well performed by our strain, which is also a *B. velezensis*, like the strains mentioned above; however, the aforementioned studies did not evaluate the parameters analyzed herein. Therefore, it is plausible to hypothesize that B.BV10 would also have applications for other cultures, such as those mentioned above.

Due to the good performance of the isolate B.BV10 in the in vitro and in vivo assays, in silico studies were carried out aiming at the identification of the strain and the mining of the genes related to the production of secondary metabolites that may have an antimicrobial activity and also information related to the isolate’s colonizing properties. One of the mechanisms that may be associated with the control of *B. cinerea* by *Bacillus* spp. is the production of lipopeptides, such as surfactins and fengycins, which are related to the antifungal activity of *B. velezensis* XT1 CECT 8661 in in vitro and in vivo assays to control *B. cinerea* [42]. The antiSMASH analysis highlighted the large diversity of molecules produced by the strain’s secondary metabolism. Among all the BGCs found, eight were responsible for synthesizing known molecules deposited in the MiBiG database, some of which were associated with the synthesis of those lipopeptides in the B.BV10 genome. This association indicates that this strain harbors the complete genetic machinery to produce these antifungal metabolites and act as a biocontrol agent with multiple targets in the pathogen.

Despite containing several clusters that have not yet been described, the *B. velezensis* strain B.BV10 showed a genetic similarity with the bacterial isolates of a high biotechnological value, as can be seen in Table 4. Clusters such as macrolactin, surfactin, bacilisyn, and bacillibactin are present and have a great similarity with the *B. velezensis* strains QST713 and FZB42, isolates present as active ingredients in Serenade^®^ and RhizoVital^®^*,* respectively [43,44]. These data corroborate the biotechnological potential of the B.BV10 strain for formulating a commercial product. Furthermore, the antagonism data against phytopathogens support the idea that some of the secondary metabolite biosynthesis gene clusters are active and confer antagonistic activity to the strain.

## 5. Conclusions

With the presented work, it possible to affirm that the *B. velezensis* B.BV10 strain presented extremely important characteristics for industrialization, being a great differential, meaning it has an easy fermentation, a simple prototype formulate, an effective control of pathogens of an agronomic importance, and biosynthetic gene clusters related to the synthesis of molecules with antifungal activity. Moreover, genes linked to the regulation/formation of biofilms, motility, and the important properties for a rhizospheric colonization were also found in the genome. The results indicates that the *B. velezensis* strain B.BV10 is a potential biocontrol agent with a plant growth-promoting ability and with important characteristics for its industrialization in the future.

## Figures and Tables

**Table 1:** Characteristics and accession number of the genomes used as reference in the comparative analyses.

Strain	GenBank Access Number	GC%
*B. velezensis* FZB42	CP000560.2	46.50
*B. velezensis* NKG1	CP024203.1	46.30
*B. velezensis* QST713	CP025079	45.90
*B. amyloliquefaciens* IT45	CP004065.1	46.60
*B. amyloliquefaciens* DSM7	NC0145511	46.10
*B. amyloliquefaciens* MT45	CP0112521	46.10
*B. subtilis* 168	NC0009643	43.50
*B. subtilis* NCIB 3610	CP020102.1	43.50
*B. cereus* FRI35	CP003747.1	35.45

**Table 2:** General characteristics of B.BV10 genome assembly according to the PGAP pipeline.

Genome Size	3,917,533 bp
GC content	46.60%
Plasmids	0
Genes (Total)	3838
Genes (Coding)	3676
rRNA operons	9
tRNA operons	7
ncRNA	5
Alignment rate	98.27%
GenBank access	NZ_CP059318.1 (https://www.ncbi.nlm.nih.gov/nuccore/NZ_CP059318.1, accessed on 1 August 2022)
BioProject	PRJNA224116 (https://www.ncbi.nlm.nih.gov/bioproject/PRJNA224116, accessed on 1 August 2022)
BioSample	SAMN15484393 (https://www.ncbi.nlm.nih.gov/biosample/SAMN15484393, accessed on 1 August 2022)

**Table 3:** Genomic comparisons of different *Bacillus* species using the OAT software for ANI% determination using OrthoANI and the Genome-to-Genome Distance Calculator (GGDC) to calculate dDDH and distance values between the genomes.

Strain	OrthoANI (%)	dDDH	Distance	GC
*B. velezensis* FZB42	99.26	94.30	0.03	0.10
*B. velezensis* NKG-1	99.06	91.70	0.07	0.27
*B. velezensis* QST713	98.43	87.40	0.09	0.67
*B. amyloliquefaciens* IT45	97.72	80.10	0.07	0.04
*B. amyloliquefaciens* MT45	94.15	55.60	0.15	0.49
*B. amyloliquefaciens* DSM7	94.04	55.60	0.17	0.49
*B. subtilis* 168	77.00	20.80	0.57	3.06
*B. subtilis* NCIB3610	77.00	20.80	0.57	3.06
*B. cereus* FRI 35	66.72	20.80	0.99	10.98

**Table 4:** AntiSMASH results showing all BGCs found within the B.BV10 genome and their positions, alongside with the best hit from the MiBiG database, relating the cluster to a possible product molecule.

Region	Type	From	To	Most Similar Know Cluster
1	lanthipeptide class II	97,265	120,306	
2	NRPS	318,617	383,476	Surfactin
3	PKS-like	929,684	970,928	Butyrosin A/Butyrosin B
4	Terpene	1,056,551	1,073,722	
5	TransAT-PKS	1,380,701	1,467,090	Macrolactin H
6	TransAT-PKS, T3PKS, NRPS	1,690,625	1,792,535	Bacillaene
7	NRPS, TransAT-PKS, Betalactone	1,866,712	2,004,072	Fengycin
8	Terpene	2,031,080	2,052,963	
9	T3PKS	2,123,567	2,164,667	
10	TransAT-PKS	2,292,697	2,386,483	Difficidin
11	NRPS	3,002,179	3,053,970	Bacillibactin
12	Other	3,580,766	3,622,184	Bacilysin

**Table 5:** Colonization-related genes.

Genes	Identity (%)	Genome Position in B. Velezensis B.BV10 (bp)		Described Function According to Subtwiki [30]
*abrB*	91.379	45,889	45,600	Transcriptional regulator of transition-state genes.
*comA*	80.380	2,996,026	2,995,395	Regulation of genetic competence and quorum sensing.
*degQ*	86.765	2,999,820	2,999,685	Stimulates production of degradative enzymes and extracellular poly-gamma-glutamate; stimulates phosphorylation of DegU by DegS.
*degU*	86.377	3,384,604	3,383,915	Two-component response regulator; regulation of degradative enzyme expression, genetic competence, biofilm formation, and capsule biosynthesis (together with SwrA); non-phosphorylated DegU is required for swarming motility.
*epsA*	99.294	3,289,519	3,288,812	Extracellular polysaccharide synthesis; putative transmembrane modulator of EpsB activity; might activate EpsB autophosphorylation and substrate phosphorylation.
*epsB*	99.853	3,288,806	3,288,126	Extracellular polysaccharide synthesis; protein tyrosine kinase; phosphorylation of EpsE.
*epsC*	98.941	3,287,880	3,286,087	UDP-N-acetylglucosamine 4,6-dehydratase; required for extracellular polysaccharide synthesis; this gene is inactive in B. subtilis 168.
*epsD*	99.649	3,286,071	3,284,932	Extracellular polysaccharide synthesis.
*epsE*	100.000	3,284,935	3,284,093	Inhibitor of motility and glycosyltransferase required for EPS biosynthesis.
*epsF*	99.296	3,284,100	3,282,964	Similar to glycosyltransferase.
*epsG*	99.547	3,282,960	3,281,857	Extracellular polysaccharide synthesis.
*epsH*	99.037	3,281,838	3,280,801	Undecaprenyl (UnDP) priming UDP-N-acetyl-glucosamine transferase; synthesis of extracellular poly-N-acetylglucosamine.
*epsI*	98.236	3,280,796	3,279,720	Glycosyltransferase; synthesis of extracellular poly-N-acetylglucosamine.
*epsJ*	98.454	3,279,723	3,278,689	UDP-N-acetyl-glucosamine transferase; synthesis of extracellular poly-N-acetylglucosamine.
*epsK*	99.605	3,278,692	3,277,175	Export of extracellular poly-N-acetylglucosamine.
*epsL*	100.000	3,277,178	3,276,570	Similar to UDP-galactose phosphate transferase; extracellular polysaccharide synthesis.
*epsM*	99.691	3,276,573	3,275,926	UDP-2,4,6-trideoxy-2-acetamido-4-amino glucose acetyltransferase; extracellular polysaccharide synthesis.
*epsN*	99.062	3,275,921	3,274,749	UDP-2,6-dideoxy 2-acetamido 4-keto glucose aminotransferase; required for extracellular polysaccharide synthesis.
*epsO*	99.793	3,274,770	3,273,805	Similar to pyruvyltransferase; extracellular polysaccharide synthesis.
*galE1*	99.899	1,173,111	1,172,119	UDP glucose 4-epimerase.
*kinA*	77.669	1,349,359	1,351,179	Two-component sensor kinase; phosphorylates Spo0F; part of the phosphorelay.
*motA*	79.044	1,317,745	1,316,930	H+-coupled MotA-MotB flagellar stator.
*motB*	76.305	1,316,958	1,316,216	H+-coupled MotA-MotB flagellar stator.
*remA*	97.778	1,565,578	1,565,847	Transcriptional regulator of the extracellular matrix genes; acts in parallel with SinR, AbrB, and DegU.
*sigD*	87.451	1,640,430	1,641,194	RNA polymerase sigma fator SigD.
*sigH*	87.931	123,748	124,385	RNA polymerase sigma factor SigH; not fully active in laboratory strains due to a mutation (V117A).
*sigW*	83.156	200,648	201,211	RNA polymerase ECF-type sigma factor SigW; required for the adaptation to membrane-active agents; activated by alkaline shock and polymyxin B, vancomycin, cephalosporin C, D-cycloserine, and triton X-100.
*sinl*	79.096	2,446,052	2,446,225	Antagonist of SinR; drives SlrR from the SlrR (LOW) to the SlrR (HIGH) state.
*sinR*	97.321	2,446,259	2,446,594	Transcriptional regulator (Xre family) of post-exponential-phase response genes.
*sipW*	100.000	2,448,076	2,447,492	Bifunctional signal peptidase I that control surface-adhered biofilm formation and processes TasA and TapA.
*spoOA*	99.251	2,412,896	2,412,096	Phosphorelay regulator; initiation of sporulation; coordinates DNA replication and initiation of sporulation by binding to sites close to the oriC.
*srfAA*	78.807	356,838	358,849	Surfactin synthetase/competence.
*srfAB*	79.825	1,892,212	1,892,099	Surfactin synthetase/competence.
*srfAC*	87.099	360,085	363,881	Surfactin synthetase/competence.
*swrA*	83.481	3,360,036	3,359,700	Master activator of flagellar biosynthesis; modulator of DegU activity; converts DegU-P from a repressor to an activator of the fla-che operon; enhances sigD transcription; controls the number of flagellar basal bodies; inactive pseudogene in strain 168.
*swrB*	81.006	1,641,222	1,641,400	Control of SigD activity; required for full SigD activity; activates the flagellar type-III secretion export apparatus by the membrane protein FliP.
*swrC*	79.906	693,419	696,562	Similar to acriflavin resistance protein.
*tasA*	99.491	2,447,427	2,446,642	Major component of the biofilm matrix; forms amyloid fibers.
*yhxB*	99.541	924,407	926,149	Alpha-phosphoglucomutase; required for UDP-glucose synthesis; inhibits FtsZ ring assembly (indirect effect due to a defect in UDP-glucose synthesis); possesses secondary phosphoglucosamine mutase activity.
*yqxM*	99.554	2,448,719	2,448,048	TasA anchoring/assembly protein.

## Data Availability

Not applicable.
